# Organelle perturbation in Alzheimer’s disease: do intracellular amyloid-ß and the fragmented Golgi mediate early intracellular neurotoxicity?

**DOI:** 10.3389/fcell.2025.1550211

**Published:** 2025-04-15

**Authors:** Lou Fourriere, Paul A. Gleeson

**Affiliations:** The Department of Biochemistry and Pharmacology, Bio21 Molecular Science and Biotechnology Institute, The University of Melbourne, Parkville, VIC, Australia

**Keywords:** Alzheimer’s disease, intracellular trafficking, Golgi apparatus, endosomes, amyloid-ß, neuron

## Abstract

Alzheimer’s disease is a devastating and incurable neurological disease. Most of the current research has focused on developing drugs to clear the extracellular amyloid plaques in the brain of Alzheimer’s disease patients. However, this approach is limited as it does not treat the underlying cause of the disease. In this review, we highlight the evidence in the field showing that the accumulation of intracellular toxic amyloid-ß could underpin very early events in neuronal death in both familial early-onset and sporadic late-onset alzheimer’s disease. Indeed, intracellular amyloid-ß, which is produced within intracellular compartments, has been shown to perturb endosomal and secretory organelles, in different neuronal models, and the brain of Alzheimer’s patients, leading to membrane trafficking defects and perturbation of neuronal function associated with cognition defects. The Golgi apparatus is a central transport and signaling hub at the crossroads of the secretory and endocytic pathways and perturbation of the Golgi ribbon structure is a hallmark of Alzheimer’s disease. Here, we discuss the role of the Golgi as a major player in the regulation of amyloid-β production and propose that the Golgi apparatus plays a key role in a cellular network which can seed the onset of Alzheimer’s disease. Moreover, we propose that the Golgi is central in an intracellular feedback loop leading to an enhanced level of amyloid-β production resulting in early neuronal defects before the appearance of clinical symptoms. Further advances in defining the molecular pathways of this intracellular feedback loop could support the design of new therapeutic strategies to target a primary source of neuronal toxicity in this disease.

## 1 Introduction

It is estimated that over 55 million people are living with dementia worldwide (World Health Organization). Alzheimer’s disease is the most common dementia and is associated with loss of memory, cognition decline, increased anxiety, impaired social interactions, and behavioral changes. Despite years of research, huge investments in private research and development funding estimated to be 42.5 billion USD since 1995 (Cummings et al., 2022), there is still no cure available to efficacy efficiently treat Alzheimer’s disease or alleviate the symptoms whilst its economic burden steadily increases in our aging society. Several monoclonal antibodies (i.e., aducanumab and lecanemab) have recently been developed to specifically target and reduce extracellular amyloid-ß plaques. These treatments are limited to a small number of patients due to the potential of serious side effects. More importantly, they failed to alleviate or revert Alzheimer’s disease symptoms, probably because the intracellular defects inherent with the initiation and maintenance of Alzheimer’s disease are still present. Moreover, the reduction in the progression of disease from these monoclonal antibody treatments was assessed from large patient cohorts and the potential benefit for any individual patient could not be readily predicted. Further work is urgently needed to understand the cellular and molecular players responsible for disease initiation that can support the development of pre-clinical diagnostic tools and novel therapeutics to treat the symptoms of Alzheimer’s disease.

**FIGURE 1:**
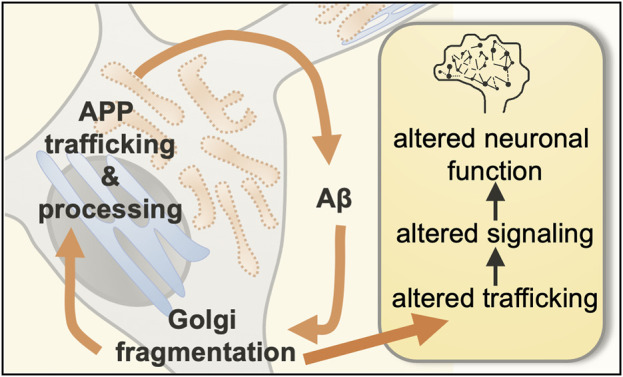
Proposed feedback loop between Golgi fragmentation and intracellular soluble amyloid-β. Neurons are composed of a cell body (or soma) as well as cellular extensions called dendrites and axons. Key early hallmarks of Alzheimer’s disease are (1) fragmentation of the Golgi apparatus and (2) enhanced levels of the neurotoxic amyloid-β peptides (Aβ). We propose that Golgi fragmentation and intracellular amyloid-β are integrated in a feedback loop involved in the regulation of neuronal trafficking, neuronal signaling, and neuronal function.

This review will focus on the intracellular perturbations observed in neuronal models of Alzheimer’s disease, notably on organellar dysfunction and the impact of intracellular amyloid-β accumulation on cellular organization, organelle structure, and membrane trafficking. We will focus on the Golgi apparatus, as the structure of this dynamic cellular hub is crucial for cell homeostasis and alterations of the Golgi structure are tightly associated with the early stage of the disease. From the collective data of published studies, we propose a working hypothesis where the Golgi apparatus perturbation, in association with perturbation of endosomal compartments, is the keystone of a very early feedback loop associated with a cascade of cellular events leading to dysfunctional cellular pathways, perturbation of cellular homoeostasis and neuronal toxicity, and which contributes to the initial seeding of Alzheimer’s disease long before the appearance of any pathological symptoms (Figure 1).

## 2 Etiology of Alzheimer’ disease

Alzheimer’s disease is a multifactorial disease associated with several histopathological hallmarks including neuronal inflammation, tau phosphorylation and neurofibrillary tangles and extracellular amyloid-ß plaques. The idea proposed by Glenner and Wong (1984) and then followed by Beyreuther and Masters (1991), Hardly and Allsop (1991), Selkoe (1991) that the accumulation of amyloid-ß is the main cause of Alzheimer’s disease is still strong (Selkoe and Hardy, 2016). Indeed, extracellular amyloid-ß (or Aß) deposition has been shown to be a central and early clinical marker of Alzheimer’s disease and has been the target of recent therapeutic monoclonal antibodies [for a recent review see (Gallego Villarejo et al., 2022)]. Amyloid-ß plaques are the result of aggregation of extracellular soluble amyloid-β produced within cells and then subsequently secreted. Soluble amyloid-β is derived from the sequential intracellular proteolytic processing of the membrane protein amyloid precursor protein (APP), by the membrane bound secretases ß-secretase (BACE1) and γ-secretase. There is considerable evidence in the literature that defects in membrane trafficking and/or cell organization contributes to aberrant amyloid-β production and accumulation within cells, leading to the neuronal dysfunction associated with Alzheimer’s disease [for reviews see (Perdigao et al., 2020; Peric and Annaert, 2015; Toh and Gleeson, 2016; Wang et al., 2024a; Wang et al., 2014)].

BACE1-mediated cleavage of APP is the first, rate limiting step, in the production of amyloid-ß; for this cleavage to take place, the transmembrane proteins BACE1 and APP need (1) to converge within an acidic compartment (Vassar et al., 1999) and (2) be incorporated into the same membrane subdomain of a given compartment for physical interaction and catalysis to occur (Fourriere and Gleeson, 2021). Membrane subdomains are generated by the self-association of different lipids to generate lipid ordered and lipid disordered domains. Hence, the regulation of intracellular trafficking as well as membrane lipid domain organization is crucial in regulating the convergence of APP and BACE1 and the production of intracellular amyloid-ß. Of relevance to Alzheimer’s disease, the *trans*-Golgi network (TGN) and early/late endosomes are compatible locations for APP processing by BACE1 (Burgos et al., 2010; Tan and Gleeson, 2019) and perturbations in the membrane organization of endosomes and the Golgi apparatus have been proposed to be intimately associated with the dysregulation of APP processing and amyloid-β production (Fourriere and Gleeson, 2021; Toh and Gleeson, 2016) (Small and Petsko, 2020). Alteration in the endosome and Golgi morphology, such as enlarged endosomes (Cataldo et al., 2004) and disruption of the Golgi ribbon structure (Haukedal et al., 2023; Joshi et al., 2015; Joshi et al., 2014) have been associated with an increase of amyloid-β level and Alzheimer’s disease. In addition, accumulation of amyloid-β represents a very early event in the etiology of Alzheimer’s disease as an increase of amyloid-ß (Aß42) can be detected in the cerebrospinal fluid (CSF) 25 years before the appearance of Alzheimer’s symptoms (Bateman et al., 2012). Indeed, in the “Jack curve,” a temporal model integrating Alzheimer’s disease biomarkers, amyloid-β is the earliest biomarker detected in the CSF and by Amyloid positron emission tomography (PET) imaging (Jack et al., 2013).

## 3 Golgi fragmentation and abnormal membrane trafficking in Alzheimer’s disease

### 3.1 Membrane trafficking in neurons

Membrane trafficking is fundamental to cellular homeostasis and function. Organelle integrity, positioning, and membrane-membrane contact sites are a few examples of the crucial cellular features which support multiple intracellular pathways. Primary neurons have unique features compared with other cell types; they are highly polarized cells composed of a cell body (or soma) surrounded by membrane extensions called axons and dendrites. To circumvent the challenge of long-distance trafficking from the soma to neurite extensions, and to allow quick responses to the environmental change, neurons have several sets of secretory organelles: in the soma (called somatic organelles), and in their neuronal extensions (called dendritic and axonal organelles). Intracellular membrane trafficking is essential to establish and maintain the neuronal network and to fine tune neural function and plasticity but the crosstalk especially between somatic and dendritic organelles in neurons is not well understood, particularly in human neurons (Wang et al., 2020).

In the aging brain and in the brain of people affected by neurological diseases (i.e., Alzheimer’s disease, Huntington disease), a decline in memory and cognition is often associated with a reduction of neuronal dendrites and axons, perturbation of organelle organization and structure, defects in membrane transport (i.e., organelle dysfunction), loss of calcium regulation and an altered signaling [for review (Castelli et al., 2019)].

Intracellular pathways have been extensively described (1) in non-polarized immortalized cells following the development of tools to study the trafficking of cargoes [for an example of tools see (Boncompain and Perez, 2013)]; in (2) rodent and *Drosophila* neurons *in vitro* [for a comparison see Wang et al. (2020)] whereas *in vivo* studies are more complex and challenging. Mouse and rat models of Alzheimer’s disease can be seen as imperfect models of Alzheimer’s disease due to non-physiological level of expression of the familial Alzheimer’s disease mutants of the human proteins (Drummond and Wisniewski, 2017) and, failure to recapitulate all aspects of the human brain. New approaches to define the intracellular organization of human neurons including human induced pluripotent stem cells (iPSC) have been developed in the last 15 years and have already made an important contribution to human neuronal biology. Human iPSC can be readily generated from accessible cell types of donor biopsies (i.e., blood cells, fibroblasts) and be differentiated into the different cell types of the brain following well-defined differentiation protocols to model Alzheimer’s disease (Penney et al., 2020).

### 3.2 Evidence of the Golgi fragmentation as a very early phenotype in Alzheimer’s disease

The Golgi apparatus is a central hub of the secretory pathway and is recognized to be a key organelle in regulating a plethora of cellular processes (Makhoul et al., 2019; Petrosyan, 2019; Wang et al., 2020). Loss of the Golgi ribbon integrity can affect membrane transport, glycosylation and signaling [for review (Li et al., 2019)]. For example, alteration of the Golgi structure perturbs APP processing and is linked to amyloid-ß production and tau pathologies (Anton-Fernandez et al., 2017a; Joshi et al., 2015).

Unique to neurons and initially described in rodent neurons is the presence of, not only a typical compact juxta-nuclear Golgi ribbon structure in the cell body, but also a specialized set of independent Golgi ministacks in dendrite extensions (Horton and Ehlers, 2003; Horton et al., 2005). The presence of highly dynamic and functional dendritic Golgi in primary human neurons has also recently been reported (Wang et al., 2023). In comparison to rodent neurons, human dendritic Golgi are more abundant (Horton and Ehlers, 2003; Horton et al., 2005; Wang et al., 2023). Dendritic Golgi structures in human neurons are a mix of punctuated and elongated structures with bi-directional dendritic movement (Wang et al., 2023). Dendritic Golgi are proposed to facilitate efficient local transport of nascent proteins to the dendritic synapses (Wang et al., 2023).

Major alterations in the morphology of the somatic Golgi is a strong indicator of changes in neuronal metabolic activity and cognition (Dubelaar et al., 2006; Martinez-Menarguez et al., 2019). Indeed, fragmentation of the Golgi cisternae has been observed in post-mortem Alzheimer’s disease brains (Baloyannis, 2014), in Alzheimer’s mouse neuronal models carrying familial Alzheimer’s disease (FAD) mutations (i.e., APPswe/PSEN1∆E9) (Joshi et al., 2014; Haukedal et al., 2023), and in a transgenic mouse model bearing mutated human Tau (P301S model) (Anton-Fernandez et al., 2017b). The structure of the Golgi apparatus is also perturbed in rat neurons stably expressing amyloid-β (Lopez et al., 2004). A recent and comprehensive study done by Haukedal et al. (2023) described abnormal Golgi morphology, and fragmentation of the *trans*-Golgi network (TGN) in human iPSC-derived neurons from patients carrying FAD mutations (either L150P or A79V PSEN1 mutation) and genetically modified human iPSC-derived neurons with the pathogenic APP Swedish mutation, using CRISPR. Golgi perturbation did not cause major changes in Golgi glycosylation in these patients’ neurons (Haukedal et al., 2023), although some glycan changes were observed in the L150P PSEN1 mutant (Haukedal and Freude, 2020). In addition, treatment of human iPSC-derived neurons with amyloid-β (Aβ42) for 24 h induces the fragmentation of the *cis* and *trans*-Golgi (Haukedal et al., 2023); while the addition of amyloid-β to primary rodent neurons also induces Golgi fragmentation (Joshi et al., 2014). The fragmentation of the Golgi by amyloid-β is a very early event observed prior to mitochondrial modification (Haukedal et al., 2023) and is likely to represent an event early in the initiation of Alzheimer’s disease [for review (Baloyannis, 2014)].

### 3.3 Abnormal membrane trafficking observed in Alzheimer’s disease

Dysregulation of membrane trafficking is observed in Alzheimer’s disease (Small and Gandy, 2006; Tan and Gleeson, 2019; Toh and Gleeson, 2016; Wang et al., 2024a) and includes defects in the trafficking of both APP and the secretases which result in elevated levels of APP processing and amyloid-β production. The TGN exports anterograde cargoes from the Golgi and receives cargoes from the endocytic pathway. Several adaptor proteins (AP) regulate trafficking at the TGN [for review see (Bonifacino, 2014)]. AP-4 notably regulates the sorting of lysosomes receptors (such as Sortlin-1) from the TGN which is important for the biogenesis of functional lysosomes in human iPSC-derived neurons (Majumder et al., 2022). The loss of AP-4 in human iPSC-derived neurons induces axon swelling due to reduction in the recruitment of retrograde transport machinery recycled from the TGN (Majumder et al., 2022). Depletion of AP-4 in HeLa cells results in delayed exit of APP from the TGN and increase APP processing (Burgos et al., 2010; Toh et al., 2017) whereas familial pathogenic mutations of APP can affect the intracellular production site of amyloid-β. Indeed, the pathogenic APP Swedish mutant is predominantly processed in the Golgi apparatus whereas wild-type APP is processed predominantly in the early endosomes (Wang et al., 2024b). Also, the membrane trafficking of other cargoes can be also altered by the disease. The axonal and dendritic transport of the brain-derived neurotrophic factor (BDNF) is altered following the addition of soluble amyloid-β oligomers to cultured primary hippocampal neurons (Gan and Silverman, 2015) or primary hippocampal neurons expressing the mutant APP Osaka (Umeda et al., 2015).

Perturbation of the intracellular distribution of the β-secretase BACE1 leads to an increase of APP processing and amyloid-β production. BACE1 trafficking is regulated by various cellular factors such as (but not limited to) the Golgi localized γ-ear containing ADP ribosylation factor binding proteins (GGAs) (Santosa et al., 2011; Toh et al., 2018), the protein sortilin (Finan et al., 2011), the retromer complex (Toh et al., 2018) [for review (Abdul-Rahman et al., 2024)], TGN adaptor protein AP1 (Tan et al., 2020), Rab proteins such as Rab11 (Buggia-Prevot et al., 2014; Udayar et al., 2013). These regulators have been intensively reviewed (Fourriere and Gleeson, 2021; Tan and Gleeson, 2019; Toh and Gleeson, 2016; Wang et al., 2024a) highlighting a key role of the membrane trafficking, the Golgi structure and the machinery controlling TGN exit of cargo in the regulation of the production of intracellular amyloid-β.

Notably, there is now clear evidence of the role of the retromer and sorting nexins in the perturbation of APP and BACE1 trafficking and amyloid-β production (Clairfeuille et al., 2016; Chandra et al., 2021; Abdul-Rahman et al., 2024). As an example, sorting nexin 27 (SNX27) is enriched in the brain and Snx27−/− mice show a developmental retardation with synaptic function defects, learning and memory defects, and a reduction of NMDA and AMPA glutamate receptors (Cai et al., 2011). These phenotypes are also common in Alzheimer’s disease highlighting a potential common role of retromer in neuronal signaling and function and in disease. Indeed, SNX27 has been shown to regulate APP trafficking and amyloid-β production in U87 cells and to colocalize with APP in primary hippocampal neurons (Lee et al., 2008).

## 4 Intracellular amyloid-ß production and its cellular effects on the organization and function of organelles in neurons

In the previous section, perturbations of membrane trafficking and more specifically the structure of the Golgi apparatus in Alzheimer’s disease were highlighted. A key question is the identity of the initial intracellular event (or events) which seed Alzheimer’s disease in human neurons. Accumulation of soluble/oligomeric amyloid-ß and the hyperphosphorylated Tau protein represent two potential early drivers of the neurotoxicity observed in Alzheimer’s disease. Both the accumulation of amyloid-β and the phosphorylation of Tau are likely to act synergistically in promoting neurotoxicity. Here, we mostly focused on the relationship between amyloid-ß and the Golgi apparatus in the context of Alzheimer’s disease. For details on the intracellular effects of Tau phosphorylation in human neurons see the recent review from Parra Bravo et al. (2024).

### 4.1 Amyloid-β production and accumulation in the cell

#### 4.1.1 Production of intracellular amyloid-β

**FIGURE 2:**
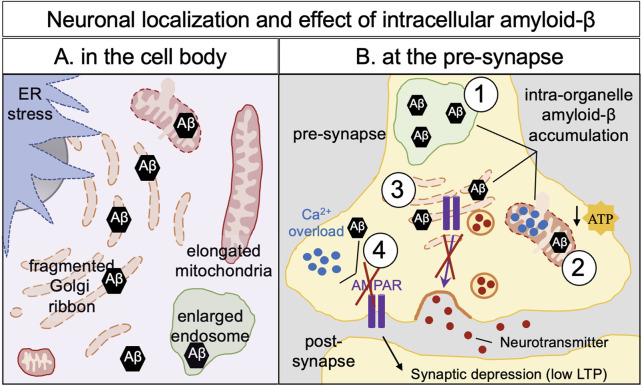
Amyloid-β neuronal localization and effects of its intracellular build-up in neurons. Intracellular defects have been observed in Alzheimer’s disease cellular models and human brain of Alzheimer’s disease: ER stress, enlarged endosomes and, fragmented, elongated and swollen mitochondria, illustrating a mitochondria dysfunction. Fragmentation of the Golgi apparatus (dispersion of the Golgi cisternae) is observed and is an early hallmark of Alzheimer’s disease which can affect protein trafficking and cellular homeostasis. **(A)** In the cell body, accumulation of amyloid-β has been observed in the mitochondria, in the early endosomes and in the Golgi apparatus leading to intraorganellar defects. **(B)** Intraorganellar accumulation of amyloid-β has been also documented in organelles in the neurites as shown here in the pre-synapse such as early endosomes (1), mitochondria (2), the Golgi apparatus (3) and in the cytoplasm (4). Accumulation of amyloid-β at the pre-synapse induced an increase of calcium storage in the mitochondria leading to a decrease of production of ATP (2), defects in post-Golgi trafficking–notably the AMPA receptors (3), overload of cytoplasmic calcium (4). Altogether, pre-synaptic amyloid-β appears responsible for a decrease in post-synaptic signaling (low LTP) and subsequently neuronal death.

The initial cleavage of APP by the β-secretase, BACE1 leads to the formation of soluble APPβ and C99 fragments, and C99 is then further cleaved by γ-secretase to produce amyloid-β. The endosomes and the Golgi apparatus are the two main intracellular sites of APP processing for amyloid-β production (Figure 2). Due to multiple γ-secretase cleavage sites on APP, several amyloid-β peptides with different length (from 37 to 41 amino acids) can be generated both in cell culture and *in vivo* (Qi-Takahara et al., 2005). Amyloid-β-40 (Aβ40) and amyloid-β-42 (Aβ42) are the major species produced; Aβ42 is more hydrophobic than Aβ40 and is more prone for self-aggregation. Indeed, extracellular Aβ42 aggregates in the brain of Alzheimer’s patients and forms the extracellular plaques (Hardy and Higgins, 1992).

A low level of amyloid-ß is produced under physiological conditions which might have several neurological roles [review (Brothers et al., 2018)]. However, the accumulation of amyloid-ß over an extended period and/or elevated levels of APP expression (and elevated levels of APP processing) are associated with Alzheimer’s disease.

Alteration of the ratio of the amyloid-β isoforms has been observed in human iPSC-differentiated neurons of Alzheimer’s patients and in human brain tissue (see Section 4.1.3). The build-up of intracellular amyloid-β42 may be the result of an imbalance from either an (1) increase of intracellular APP processing and amyloid-β production, (2) increase of cell uptake of extracellular amyloid-β, or (3) defect in the clearance of intracellular amyloid-β.

#### 4.1.2 Toxicity of the intracellular amyloid-β

Although a long-time focus in the field has been the extracellular aggregates of amyloid-β, the potential toxicity of the extracellular amyloid-β plaques is unclear. Firstly, the number of amyloid-β plaques does not seem to be linked with the severity of patient’s symptoms (Lue et al., 1999; McLean et al., 1999), whereas the level of soluble amyloid-β is linked with the severity of clinical symptoms and the loss of synapses (Lue et al., 1999). Secondly, Alzheimer’s mouse models accumulate intracellular amyloid-β, synaptic dysfunction (Oddo et al., 2003) and cognitive defects (Billings et al., 2005), without or with very little extracellular amyloid-β deposits. Moreover, cognitive defects in these mouse models can be restored after the removal of intracellular amyloid-β (Billings et al., 2005). Thirdly, monoclonal antibodies used clinically which target the extracellular plaques might clear the extracellular amyloid plaques, as observed on PET scans (Klein et al., 2019; Rinne et al., 2010; Sevigny et al., 2016) but still failed to restore the cognitive defects of patients or to alleviate Alzheimer symptoms [see the review (van Dyck, 2018)] indicating that extracellular amyloid-β plaques are not the solely responsible for neuronal toxicity. In addition, therapeutic application of these monoclonal antibodies does not appear to influence familial Alzheimer’s disease patients treated prior to the presentation of the symptoms (DIANE 2020 study).

There is considerable evidence supporting the proposal that soluble amyloid-β could be even more toxic than the amyloid-β plaques, which may rather represent an inactive waste reservoir of aggregated amyloid-β [for general reviews (Haass and Selkoe, 2007; LaFerla et al., 2007)]. The familial pathogenic E693D APP mutation (also known as APP Osaka) illustrates this point. APP Osaka leads to the production of an amyloid-β product that can oligomerize but cannot aggregate; patients carrying this mutation have an increased level of intracellular amyloid-β but develop only few extracellular amyloid-β plaques (Tomiyama and Shimada, 2020). In primary mouse neurons, the expression of APP Osaka leads to the accumulation of intracellular amyloid-β which is accompanied by a reduction of spines and an alteration of the transport of brain-derived neurotrophic factor (BDNF), mitochondria and recycling endosomes (Umeda et al., 2015).

Soluble amyloid-β is linked with synapse defects and impairment (Bayer and Wirths, 2010; Gouras et al., 2010), neuroinflammation, cellular stress and cellular plasticity (Gallego Villarejo et al., 2022). The accumulation of amyloid-β42 in pre- and post-synaptic multivesicular bodies (MVB) in mouse, rat, and human neurons is associated with abnormal synapse morphology, before the appearance of extracellular amyloid-β plaques in animal models (Takahashi et al., 2002).

Another important observation is that soluble amyloid-β dimer from human brain extract, but not aggregated amyloid-β, can perturb synaptic physiology (i.e., long-term potentiation or LTP, learning behavior) in rodents (Shankar et al., 2008). As intraneural soluble amyloid-β is observed in the human brain before the appearance of extracellular amyloid-β plaques and tau phosphorylation, it is likely that there is a build-up of the soluble intraneural pool prior to the appearance of classical hallmarks of Alzheimer’s disease (Welikovitch et al., 2018). In conclusion, there is a plethora of evidence *in vitro* and *in vivo* showing that accumulation of amyloid-β in the brain is a driver of neurotoxicity (Haass and Selkoe, 2007; Hardy and Selkoe, 2002).

#### 4.1.3 Evidence that FAD mutations increase the production of amyloid-β

FAD-associated mutations in APP or in PSEN1/PSEN2 are associated with an increase of intracellular amyloid-β (Aβ42) (Qi-Takahara et al., 2005; Suarez-Calvet et al., 2014). Analysis of immunoprecipitated amyloid-β peptides from human iPSC-neuronal supernatants by mass spectrometry has shown that the ratio Aβ42/Aβ40 was raised in almost all cells carrying FAD mutations. One exception is the pathogenic FAD APP mutation London (APP V717I) where one study reported that the ratio Aβ38/Aβ40 was increased (in 100 days post neural induction neurons) (Arber et al., 2020), although another study using younger (40–50 days) iPSC-derived neurons did observe an increase in Aβ38/Aβ40 and in the Aβ42/Aβ40 ratio (Muratore et al., 2014). In addition, the PSEN1 E280G mutation in human iPSC-derived neurons is associated with an increase of Aβ43 (Willumsen et al., 2023). Therefore, mutations in APP and/or enzyme responsible for APP processing are associated with changes in the level and identity of the amyloid-β products, which may be an important factor in the early onset of Alzheimer’s disease.

#### 4.1.4 Import of extracellular amyloid-β

Amyloid-β is generated in intracellular compartments and then secreted by the cells, leading to an accumulation of amyloid-β in the extracellular environment. Extracellular amyloid-β can then be (re-) internalized by cells using various pathways such as ion channels (Arispe et al., 1993; Bhatia et al., 2000; Kawahara et al., 2009), membranes pores (Inoue, 2008), fluid phase macropinocytosis (Mandrekar et al., 2009), binding receptors (i.e., RAGE, NMDAR, nACHR, FcgammaRIIb, PirB, PrPc, EphB2) and/or lipids and cholesterol (Kanekiyo et al., 2011; Williamson et al., 2008) on the cell surface which are then internalized. Interaction between extracellular amyloid-β and membrane receptors can induce intracellular toxicity mediated by processes such as Golgi fragmentation, ER stress and mitochondria dysfunction [for a review see Kam et al. (2014)] and could lead to a vicious cycle of increase of intracellular production of amyloid-β.

#### 4.1.5 Mechanisms of intracellular amyloid-β clearance

The secretion of intracellular amyloid-β can reduce the load of the toxic amyloid-β within neurons. Indeed, inhibition of exocytosis in the neuroblastoma cell line, SH-SY5Y, results in the intracellular accumulation of amyloid-β (Zheng et al., 2012). Subsequent clearance of extracellular amyloid-β can take place by cellular uptake and lysosomal degradation (Baranello et al., 2015). Microglia and astrocytes are important non-neuronal cells of the brain in controlling amyloid-β accumulation. However, both cell types are also responsible for dispersal of amyloid-β and inducing an inflammatory response resulting in the progression of the disease [for reviews see (Avila-Munoz and Arias, 2014); (Webers et al., 2020)] inflammation mechanisms notably trigger fragmentation of the Golgi. In Alzheimer’s disease, activated astrocytes could have a role in disease propagation (Kumar et al., 2023) and in amyloid-β production (Zhao et al., 2011). Human iPSC-derived astrocytes have been shown to store high levels of amyloid-β (Konstantinidis et al., 2023). In addition to its secretion, intracellular soluble amyloid-β can also be cleared by other mechanisms. As an example, ESCRT proteins have been shown to limit intracellular amyloid-β accumulation by targeting amyloid-β to the lysosome for degradation on N2a-APP cells (Edgar et al., 2015).

#### 4.1.6 Accumulation of intracellular amyloid-β is a very early event in Alzheimer’s disease

Perturbation of amyloid-β production, import and clearance could be associated with the early events of Alzheimer’s disease and intracellular accumulation of amyloid-β might have a role in the cognitive defects observed in Alzheimer’s disease. An integrated model of the time course of Alzheimer’s biomarkers (which rely on clinical detection capability) has been proposed and is known as the “Jack curve” (Jack et al., 2013). In the Jack curve, amyloid-β is the first biomarker detected in the CSF, and before the development of cognitive defects. This observation highlights the importance of amyloid-β very early in the development of Alzheimer’s disease; however, to be able to understand the seeding of Alzheimer’s disease, human neurons need to be characterized at a much earlier time point than currently performed. Also, studies investigating clinical approaches to treat Alzheimer’s disease need to have a greater focus on the “intracellular amyloid-β hypothesis” which describes the “ultra-early-stage pathology of Alzheimer’s disease” [to read more about the “ultra-early phase pathology of Alzheimer’s disease see (Okazawa, 2021)]. Another point to consider is the clinical definition of healthy or control donor/patients versus Alzheimer’s disease patients. Usually, a “healthy donor” refers to a person who has not developed Alzheimer’s clinical symptoms (i.e., no cognitive defect, no amyloid-β plaques detected). But the diagnostic tools are unable to identify whether the intracellular levels of amyloid-β are increased and have initiated disease onset in this designated “healthy control” group.

### 4.2 Intracellular toxicity of soluble amyloid-β in neurons

#### 4.2.1 Effect of intracellular amyloid-β on neuronal function

##### 4.2.1.1 Neuronal morphology

There is evidence that intraneural accumulation of the processed products of APP, together with phosphorylated tau, affects synaptic function leading to the cognitive defects associated with Alzheimer’s disease [for reviews (Bayer and Wirths, 2010; Spires-Jones and Hyman, 2014)]. Accumulation of intracellular amyloid-β contributes to synaptic dysfunction (Edwards, 2019; Selkoe and Hardy, 2016) (Figure 2), whereas low concentration of intracellular amyloid-β has been shown to have a positive effect on modulating memory and synaptic plasticity. A reduction of the size of dendritic spines and a modification of spine morphology are observed after intracellular accumulation of amyloid-β in rodent cortical neurons (Kienlen-Campard et al., 2002; Ochiishi et al., 2019) and a reduction of synaptic density has been observed in human iPSC-derived neurons carrying the pathogenic PSEN1 mutations (L150P and A79V) (Haukedal et al., 2023). Addition of amyloid-β (Aβ42) to human iPSC neural stem cells encapsulated in 3D gels impairs cell plasticity, neurogenic ability and their ability to form a network (Papadimitriou et al., 2018).

##### 4.2.1.2 Neuronal activity

There is a nexus between elevated levels of intracellular amyloid-β and neuronal hyperexcitability. Although a role for amyloid-β (and Tau) in synaptic dysfunction in Alzheimer’s disease has been reported, the precise underlying mechanisms between neuronal activity and amyloid-β is not well understood. Indeed, the molecular connection between amyloid-β and synaptic activity is probably multifactorial and non-linear [for a recent modelling of soluble amyloid-β dependent neuronal hyperactivation in early Alzheimer’s disease, see Bonifazi et al., (2024)]. Increased brain activity is observed very early in Alzheimer’s patients [for an example see (Dickerson et al., 2005)] and increased neuronal excitability is also observed in a rodent model of Alzheimer’s disease (*APdE9* mice carrying the APP Swedish mutation and a deletion of exon 9 of PSEN1) compared to wild-type rodents and is linked with the accumulation of amyloid-β and an enhanced susceptibility of seizure and epileptiform activities (Minkeviciene et al., 2009). Human intracellular amyloid-β has been reported to increase synaptic transmission, neuronal excitability, and neuronal synchronization *in vitro* and *in vivo* in hippocampal models with impaired inhibitory and excitatory synaptic transmission (Fernandez-Perez et al., 2021). As an example, human iPSC-derived neurons from Alzheimer’s patients (PSEN1 exon 9 deletion; PSE_M146V_ mutation; APP_SWE_ mutation) show an enhanced electrical activity compared to their respective isogenic control neurons in 2D culture and in 3D organoids which is inversely correlated with the length of their neurites in 2D (Ghatak et al., 2019). Supplementation of amyloid-β (50 nM) within the mouse prefrontal cortex (anterior cingulate cortex) induced an hyperexcitability of excitatory pyramidal cells (Ren et al., 2018).

Excess amyloid-β is also linked with perturbation of synapse function; for a review on the neurotoxic role of amyloid-β in synaptic long-term depression and the linkage with NMDA receptor trafficking, see (Guntupalli et al., 2016). The addition of amyloid-β (from a cell culture conditioned media) to human iPSC-derived cortical neurons alters the spontaneous neuronal activity with a decrease in its amplitude and frequency (Nieweg et al., 2015). Also, the addition of amyloid-β is followed by a loss of axonal vesicle clusters and the loss of postsynaptic AMPA receptors, highlighting a role of amyloid-β in axonal transport of synaptic vesicles (Nieweg et al., 2015). On the other side, human and synthetic intracellular amyloid-β oligomers have been reported to increase neuronal excitability (or neuronal firing) by regulating synaptic activity through AMPA receptors (Fernandez-Perez et al., 2021). And a passive diffusion of oligomerized amyloid-β into neurons induces also a rapid synaptic response and enhancement of AMPA receptor mediated synaptic transmission (Whitcomb et al., 2015). Extended treatment (8 days) of human iPSC-derived neurons with amyloid-β (present in the supernatant from CHO cells carrying the familial pathogenic APP_V717F_ mutation) lead to alterations in the organization of axonal vesicle clusters and impaired the postsynaptic AMPA receptors (Nieweg et al., 2015). Chronic treatment of human iPSC-derived neurons using a physiological concentration of 10 nM of amyloid- β is associated with defects in neuronal firing but without inducing cellular death (Berry et al., 2018). In primary mouse cortical neurons (pathogenic APP SWE model), soluble amyloid-β reduces the localization of NMDA receptors at the plasma membrane by promoting NMDA endocytosis at the synapses (Snyder et al., 2005). However, Cirrito et al. demonstrated that synaptic activity *in vivo* regulates extracellular amyloid-β in brain interstitial fluid (ISF) using microdialysis in mouse’s brain (Cirrito et al., 2005) and that an increase of synaptic activity can lead to an elevation of APP endocytosis which increases amyloid-β production *in vivo* (Cirrito et al., 2008), starting a deleterious feedback loop.

It is important to mention that a decrease of amyloid-β production (due to an increase of alpha-secretase cleavage and C83 production thereby limiting amyloid-β production) has been observed following neuronal activation (by NMDA receptor activity) in primary cortical mouse neurons (Hoey et al., 2009). Indeed, a pulse of NMDA, leading to neuronal activation, drives the trafficking of synapse-associated protein-97 (SAP97) and ADAM10 (alpha-secretase) to the synapse, which promotes a non-amyloidogenic APP processing following neuronal activation (in mouse brains, hippocampal primary mouse neurons and COS-7 cells) (Marcello et al., 2007).

Overall, the conflicting results on the effect of amyloid-β on primary neurons activity highlight the complex interconnection between amyloid-β and neuronal function. Kamenetz et al. (2003) have shown using mouse organotypic hippocampal slices that neuronal activity can modulate APP processing and amyloid-β production, whereas amyloid-β can modulate neuronal activity. Therefore, it has been proposed that both amyloid-β and neuronal activity are part of a regulatory loop (Kamenetz et al., 2003), and see Palop and Mucke (2010) for a dated but comprehensive review (Palop and Mucke, 2010). However, the mechanism responsible is poorly understood.

Finally, Li et al. (2013) observed two distinct responses in cultured primary culture neurons following treatment with a chemical neuronal stimulator (using 100 μM picrotoxin): 1) an increase in the production and secretion of amyloid-β, 2) followed by negative/protective feedback associated with a decrease of amyloid-β production overtime (Li et al., 2013). Collectively, these studies also indicate that the intracellular concentration of amyloid-β is crucial to the outcome, in particular, changes in the amyloid-β intracellular concentration can drive opposite cellular effects. Overall, amyloid-β has multiple effects on neuronal networking, synaptic responses and contributes to neurotoxicity.

#### 4.2.2 Effect of intracellular amyloid-β on the cytoskeleton and Tau protein

Tau is a microtubule-associated protein in neurons required for the polymerization and bundling of microtubules (MTs) polymerization and bundling and is associated with neurodegeneration and Alzheimer’s disease. Tau is also able to bind actin filaments and can act as molecular linker between actin and microtubules (Elie et al., 2015). There is evidence that intracellular amyloid-β can induce the hyperphosphorylation of tau, leading to a disruption of the MTs network in axons and dendrites and perturbation of local trafficking. Indeed, amyloid-β oligomers induces the mislocalization of Tau in the soma and the dendrites of primary neurons, depletion of spines, an increase of Ca^2+^ and a perturbation of the microtubules (Zempel et al., 2013; Zempel et al., 2010). The addition of extracellular amyloid-β to human iPSC-derived neurons has been shown to be associated with an increase of the phosphorylation of tau protein (Nieweg et al., 2015); also the expression of intracellular amyloid-β fusion protein (with GFP) in primary mouse neurons is associated with an increase of Tau phosphorylation and alteration of the spine morphology (Ochiishi et al., 2019). Hence the pathways of amyloid-β production and hyperphosphorylation of Tau are interconnected.

## 5 Proposed hypothesis: the Golgi and amyloid-β at the center of a feedback loop in Alzheimer’s disease

The Golgi apparatus is a highly dynamic organelle, and its function is essential in many key cellular pathways (i.e., trafficking, glycosylation, mitosis, apoptosis, cellular stress, autophagy, cell division). The contribution of the Golgi to a wide range of cellular processes is underpinned by an extensive set of signaling genes and pathways wired into the Golgi as identified from genome wide screens (Kondo et al., 2017). In addition, perturbation in the dynamics and/or architecture of the Golgi apparatus is associated with numerous defects and diseases [cancer, neurodegeneration, immunological disorders) (see recent reviews (Choi et al., 2022; Makhoul et al., 2019; Zobaroglu-Ozer and Bora-Akoglu, 2024)].

Importantly, the form and function of the Golgi apparatus are interconnected and interdependent [see an example developed in the review (Petrosyan, 2019)]. The Golgi ribbon can exist as a tightly packed compact structure, an expanded ribbon morphology, or can be fragmented into numerous segments. The compaction of the Golgi apparatus is notably controlled by golgin proteins (i.e., GM130, GRASP65, giantin) and the cytoskeleton (actin and microtubules). The Golgi is a trafficking hub for anterograde and recycling transport [for review (Saraste and Prydz, 2019)] and fragmentation of the Golgi has been observed in cellular and rodent models of Alzheimer’s disease and in brains of Alzheimer’s patients. The loss of Golgi functions can exacerbate an underlying pathological condition [i.e., in Alzheimer’s disease (Baloyannis, 2014; Haukedal et al., 2023; Martinez-Menarguez et al., 2019; Stieber et al., 1996)]. However, the effect of the fragmentation of the Golgi on the normal regulation of membrane flow between anterograde and recycling pathways is not well described; yet is critical for protein transport to a variety of destinations and for endosomal-lysosomal homeostasis.

### 5.1 Golgi fragmentation and its cellular consequences

The loss of the Golgi ribbon structure is often described as “fragmented,” meaning that the Golgi stacks forming the Golgi ribbon are disjointed. Golgi fragmentation is observed in physiological processes such as cell cycle/mitosis, migration, polarization. This fragmentation is reversible as the Golgi ribbon can be reformed shortly after the cell division [for reviews (Colanzi and Sutterlin, 2013; Rajanala et al., 2021)]. However, irreversible fragmentation of the Golgi is commonly reported in the very early stages of neurodegenerative diseases [see the overview in the Editorial by Rabouille and Haase (Rabouille and Haase, 2015; Martinez-Menarguez et al., 2019)]. As Golgi fragmentation is an early event in the Alzheimer’s disease cascade, it could represent a new cellular target for early therapeutic intervention supported as the restoration of a neuronal compact Golgi ribbon structure protects from disease progression in animal models (Joshi et al., 2015; Joshi et al., 2014).

**FIGURE 3:**
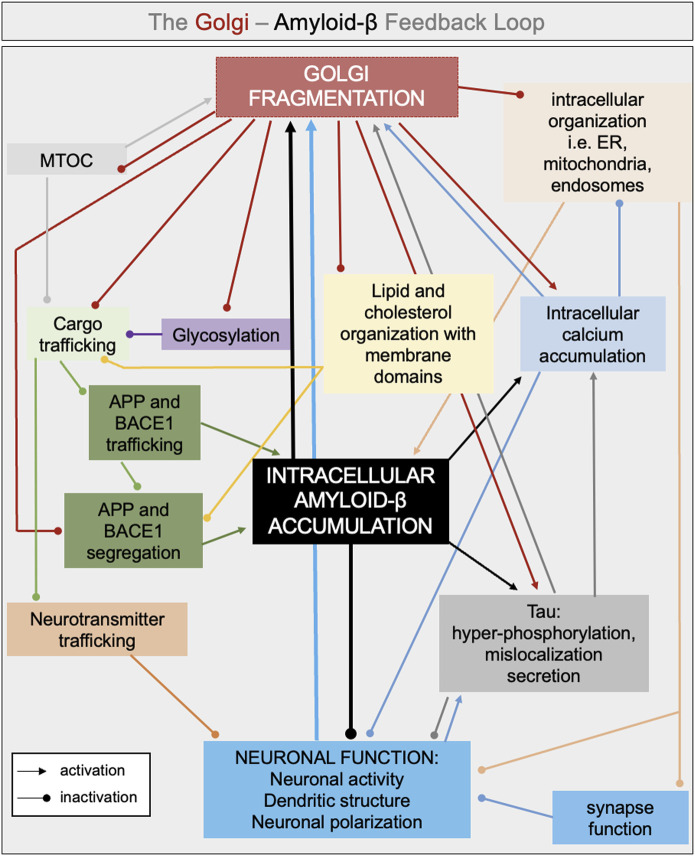
Interconnected pathways involved in the intracellular feedback loop between Golgi fragmentation and amyloid-β. Golgi fragmentation and accumulation of intracellular amyloid-β are proposed to be in the middle of a feedback loop leading to defects in neuronal activity, neuronal polarization and neuronal function. Several interconnected pathways can lead to either an increase of amyloid-β production and/or accumulation and in Golgi fragmentation such as: defects in glycosylation, defect in cargo trafficking (i.e., neurotransmitter, APP, BACE1), changes in lipid and cholesterol organization with membrane domains, increase in intracellular and intraorganellar calcium. The color chosen for each arrow reflects the source of the defects (i.e., black arrow if the intracellular defect is led by the accumulation of amyloid-β).

An early event involving fragmentation of the Golgi ribbon could have several outcomes that contribute to the progression of Alzheimer’s disease, as follows and in Figure 3.

#### 5.1.1 Increase of intracellular amyloid-β production

Wild-type APP and BACE1 are well segregated through the Golgi ribbon in HeLa cells and primary mouse neurons; possibly to prevent APP processing by BACE1 (Fourriere et al., 2022). In addition, the Golgi apparatus has been shown to be the main site for the pathogenic SWE-APP processing and the production of amyloid-β in HeLa cells (Wang et al., 2024b). Fragmentation of the Golgi could restrict APP and BACE1 intra-Golgi trafficking and their TGN exit. Therefore, the resulting concentration of APP and BACE1 in the dispersed Golgi cisternae will increase the probability for APP to colocalize with BACE1, resulting in an increase of amyloid-β production in the Golgi. Because the fragmentation of the Golgi, induced by the depolymerization of microtubules following nocodazole treatment, does not affect the segregation of wild-type APP and BACE1 in the small Golgi units (Fourriere et al., 2022), we hypothesize that other factors play a role in the production of amyloid-β when the Golgi is fragmented such as pathogenic APP mutations and cholesterol and saturated lipid membrane contents.

#### 5.1.2 Cascade of events and cellular disorganization

As the Golgi apparatus is a central organelle, fragmentation of the Golgi ribbon can impact on other intracellular organelles (i.e., mitochondria, ER, endosomes, lysosomes) leading to multiple cellular defects and cellular disorganization, driving a vicious cycle leading to an increase in amyloid-β production. The Golgi interacts with organelles via membrane- membrane contacts. Membrane contacts are important mediators of a range of cellular processes and dysfunctional membrane contact sites has been linked to neurological diseases (Prinz et al., 2020). As membrane contacts are dependent on the spatial organisation of organelles, it is likely that the loss of the Golgi ribbon and dispersal of Golgi stacks will result in disruption of these contact sites. In addition, as the Golgi is responsible for sorting and delivery of other organelle proteins (i.e., lysosomal enzymes to the lysosomes) (Majumder et al., 2022), disruption of the Golgi ribbon could retard cargo trafficking to their destination (i.e., the endosomal pathway) and thereby perturb numerous intracellular organelles and cellular functions (i.e., lysosomal function and autophagy) (Riederer et al., 1994).

##### 5.1.2.1 Mitochondria

The Golgi apparatus has been shown to influence mitochondrial fission which is driven by phosphatidylinositol-4-phosphate (PI(4)P) containing vesicles derived from the TGN (Rasmussen et al., 2020). PI(4)P levels are likely to be altered following a fragmentation of the Golgi, as discussed below. Mitochondrial dysfunction and Golgi fragmentation has been recently linked in asthma. Indeed, induced inflammation of the pulmonary epithelial has been shown to induce Golgi fragmentation through the ULK1/Atg9a/Rab9 pathway and is associated with mitochondrial oxidative stress (Xu et al., 2024). Interestingly, Rab9 facilitates vesicular trafficking between late endosomes and the TGN (Lombardi et al., 1993) and is also localized in mitochondria (Xu et al., 2024). However, the potential role of Rab9 in the Golgi fragmentation, inflammation and mitochondria defect in human neurons still needs to be tested.

Imbalanced intracellular calcium and a reduction in glucose metabolism indicating a potential role of mitochondria in Alzheimer’s disease, have been linked with the early stages of the disease (Minoshima et al., 1997). APP and amyloid-β have been observed in the mitochondria and in the Mitochondria-Associated-Membrane in Alzheimer’s disease patient’s brain (Devi et al., 2006), [for review see (Pavlov et al., 2009)]. Amyloid-β (Aβ40 and Aβ42) has been found in mitochondria extracts from the mouse cortex (Manczak et al., 2006). The accumulation of amyloid-β in mitochondria is observed before the appearance of extracellular amyloid-β plaques and increases with aging. Changes in the intracellular calcium concentration, which can be triggered by Golgi fragmentation, has been shown to affect the mobility and plasticity of mitochondria which is tightly linked with synapse plasticity [for a recent review see (Sayehmiri et al., 2024)]. Fragmentation of the Golgi could also indirectly perturb the structure, dynamics and functions of mitochondria, for a recent review see (Strope and Wilkins, 2024), or mitophagy, which can collectively lead to a redistribution of the APP and amyloid-β pools present in mitochondria.

In Alzheimer’s disease, alterations of mitochondria morphology, distribution, dynamic motility, inter-organelle membrane interactions and contact sites with the endoplasmic reticulum (ER), and quality control have been observed in human iPSC-derived neurons from patients with Alzheimer’s disease [for review (Chen and Chan, 2009; Trombetta-Lima et al., 2021)]. Mitochondrial dysfunction is also observed in human iPSC-derived neurons bearing FAD mutations such as in APP, or in presenilin 1 and 2 (subunit of the γ-secretase: PSEN1, PSEN2) [as listed in the recent review Trombetta-Lima et al. (2021)]. A reduction of mitochondrial mobility has been observed in neurons expressing heterologous APP (Gao et al., 2020).

ER-mitochondria contact sites (MAM) (Wu Y. et al., 2017) have an important role in signal regulation in neuronal cell bodies, axons, dendrites and synaptic regions (Hirabayashi et al., 2017; Leal et al., 2018; Leal et al., 2020) notably by regulating calcium homeostasis (Wu S. et al., 2017), which is often perturbed in Alzheimer’s disease (Lee et al., 2018) [for reviews see (Area-Gomez et al., 2018; Calvo-Rodriguez and Bacskai, 2021; Lim et al., 2021; Markovinovic et al., 2022)], as well as cholesterol intake (Montesinos et al., 2020), and are potential sites of amyloid-β production (Schreiner et al., 2015).

##### 5.1.2.2 Endoplasmic reticulum

Due to their closed proximity and related cellular functions, notably with the ER-exit sites, it is more likely that fragmentation of the Golgi apparatus will also directly affect ER morphology and functions. Integrity of the ER-Golgi contact sites are required for lipid transfer and for controlling the levels of PI(4)P at the TGN in non-polarised HeLa cell (Venditti et al., 2019). Indeed, morphological changes of the ER structure have been described in aging and various neurological diseases including Alzheimer’s and Parkinson diseases [for review (Sree et al., 2021)]. For example, the organization of the ER tubules is impaired in the axon of brains of Alzheimer’s mouse models and Alzheimer’s patients (Sharoar et al., 2016). In addition, exogenous amyloid-β treatment of human iPSC-derived neurons results in ER stress and synaptotoxicity (Nieweg et al., 2015).

The E4 allele of apolipoprotein E (*ApoE4*), a risk factor for Alzheimer’s disease, increases ER-mitochondria communication and activity in human fibroblast and primary mouse neurons, which could contribute to the disease (Tambini et al., 2016). APOE has been found in mitochondria and in the MAM in human cultured hepatocytes and interacts with the Mitochondrial Import Receptor Subunit TOM40 and the transporter of small ions VDAC1 (Voltage Dependent Anion-Selective Channel 1) (Rueter et al., 2024), which can link organelles together to generate organelles complexes. This finding is interesting as VDAC1 can potentially traffic through the Golgi apparatus (Buettner et al., 2000); and its trafficking will be affected by Golgi fragmentation. Moreover, VDAC1 is involved in Ca^2+^ homeostasis between the ER and the mitochondria in fragile X syndrome patients iPSC-derived neurons (Geng et al., 2023) and in mitochondrial defects through its interaction with phosphorylated-Tau (Vijayan et al., 2022); demonstrating a role in calcium homeostasis and neuronal function. Future studies to investigate the localization of APOE and VDAC1 in healthy human neurons and human neurons from Alzheimer’s patients will be important to better understand the nexus between APOE, VDAC1 and the Golgi apparatus.

##### 5.1.2.3 Endosomes

The Golgi, especially the TGN, is a cellular hub interconnecting the synthetic and recycling pathways (Chia et al., 2013a). Endosomes are dynamic sorting organelles supporting the fast internalization, recycling, and/or degradation of pre- and post-synaptic membrane proteins. Early and recycling endosomes and late endosomes/lysosomes have been identified in both the soma and the neurites of human iPSC-derived neurons (Boecker et al., 2020; Wang et al., 2023). Endocytosis mediates the recycling of plasma membrane proteins from the early endosomes, either directly or indirectly via the TGN, or their degradation by the transport along the early and late endosomes to lysosomes. Therefore, fragmentation of the Golgi apparatus or perturbation of organization of the TGN could affect the endocytosis and recycling of pre- and post-synaptic membrane proteins, directly affecting neuronal function. Early endosomes are 2.5-fold larger in post-mortem human brain tissues of clinically diagnosed individuals with Alzheimer’s disease compared to neurologically symptom-free individuals (Cataldo et al., 1997) whereas aberrant swollen early endosomes have been observed in human iPSC-derived neurons with or without Alzheimer’s disease risk factors (Cataldo et al., 2000; Haukedal et al., 2023; Hung and Livesey, 2018; Israel et al., 2012; Kim et al., 2016; Kwart et al., 2019; Lin et al., 2018; Woodruff et al., 2016). These morphological changes could be the result of an accumulation of endocytic material, including amyloid-β, or changes in the balance of anterograde and retrograde transport between the TGN and the early endosomes.

Importantly, early endosomes are one of the major intracellular sites for amyloid-β production and perturbation of the endocytic pathway is strongly associated with Alzheimer’s disease before the deposition of aggregated amyloid-β [for review (Nixon, 2017; Small et al., 2017; Cataldo et al., 2000)] and changes in the expression of endocytic trafficking genes are associated with a risk of Alzheimer’s disease (Karch and Goate, 2015).

Studies have shown that endosomal pathologies in the brain and in cellular models is independent of amyloid-β peptides (Hung and Livesey, 2018; Jiang et al., 2010; Kim et al., 2016; Woodruff et al., 2016; Kwart et al., 2019). However, the functional impact of elevated intracellular amyloid-β and Golgi fragmentation on the organization of the Golgi-endosome hub, required for optimal neuronal function, is still underdefined.

#### 5.1.3 Trafficking

Fragmentation of the somatic Golgi ribbon is very likely to result in trafficking defects in the dendrites and axons, such as defects in neurotransmitter trafficking to the synapse resulting in the perturbation of synapse and neuronal function. In rodent primary neurons, perturbation of the Golgi structure following GM130 knockdown results in defects in polarization of the dendrites (Huang et al., 2014) which could be the result of membrane trafficking perturbations. Of relevance is that Golgi fragmentation is known to slow down anterograde trafficking of cargo in non-polarized cells (Lee et al., 2012), hence there may be an alteration in the kinetics of cargo transport in neurons with a fragmented Golgi. Also, fragmentation of the Golgi is likely to affect the trafficking of APP and the segregation of APP from the secretases, and thereby result in dysregulated APP processing (Fourriere et al., 2022; Fourriere and Gleeson, 2021; Wang et al., 2024a).

#### 5.1.4 Glycosylation

Golgi fragmentation can result in alterations in the organization of glycosylation enzymes with individual Golgi compartments. Glycosylation is important in cargo trafficking to the apical and basolateral surface membranes in polarized endothelial and epithelial cells and is also likely to be important for the polarized trafficking of cargoes in primary neurons. A recent paper from Nakano’s group has shown that the Golgi ribbon, in non-polarized cells, contains small Golgi units or domains of glycosylation enzymes which might work independently from each other (Harada et al., 2024). Perturbation of the Golgi ribbon has been shown to affect glycosylation (Puthenveedu et al., 2006), and, in addition, the loss of the ribbon has been shown to disrupt organized zones of glycosyltransferases and affect their functional activity (Harada et al., 2024). Moreover, neurons are recognized to be very sensitive to changes in glycosylation (Sato and Kitajima, 2021) and alteration of N-glycosylation has been observed in Alzheimer’s patients (Zhang et al., 2020). In human primary neurons, dendritic Golgi are functional small functional Golgi units able to receive cargoes from the endoplasmic reticulum (Wang et al., 2023). Further work is needed to define the glycosylation events that are mediated by these Golgi outposts and the impact of morphological perturbation of the somatic Golgi in Alzheimer’s disease.

#### 5.1.5 Calcium storage

The Golgi is a storage pool for calcium and magnesium regulated by actin cofactors (von Blume et al., 2011). Perturbation of the structure of the Golgi can alter calcium/magnesium storage, resulting in a perturbation of the intracellular pH and signaling pathways which could have major consequences on neuronal signaling [for reviews (Li and Wang, 2022; Martinez-Menarguez et al., 2019)].

### 5.2 Possible molecular driver(s) for Golgi fragmentation in primary neurons?

The molecular mechanism and sequence of events associated with the loss of the Golgi ribbon structure in Alzheimer’s disease are not well defined but probably include a complex network of signaling pathways, small G protein, cytoskeleton and kinases which can regulate a number of different pathways which all convert the Golgi ribbon into different morphologies (Derby et al., 2007; Farhan and Rabouille, 2011; Goud and Gleeson, 2010; Houghton et al., 2012; Kulkarni-Gosavi et al., 2019; Luini and Parashuraman, 2016; Mayinger, 2011). The precise functional consequence of the loss of the ribbon is likely to depend on which pathways are activated in the disease.

#### 5.2.1 Membrane transport and Golgi scaffolds

Perturbation of the balance of anterograde and recycling membrane transport pathways will alter membrane flow to and from the Golgi and could result in loss of the normal morphology of the somatic Golgi in neurons. Components of the endosome-Golgi retrograde pathway are risk factors in Alzheimer’s disease (Karch and Goate, 2015). For example, recent studies have shown a crucial role for the retromer complex and the endosomal cargo receptor, SORLA, in the endosomes-to-TGN/Golgi recycling pathway, and might contribute to Alzheimer’s disease (Abdul-Rahman et al., 2024; Small, 2008; Small and Petsko, 2015). Furthermore, the retromer component, VPS35, and a sortilin-related protein are involved in the retrograde transport of BACE1 and APP to the TGN (Okada et al., 2010; Rogaeva et al., 2007). Enlarged endosomes have been observed in Alzheimer’s disease, together with an accumulation of APP, and endosome enlargement may also result in perturbation of Golgi morphology by reduced membrane flow from defects in retrograde transport (Okada et al., 2010; Rogaeva et al., 2007).

GRASP proteins play a major role in regulating the architecture of the Golgi (Zhang and Wang, 2020) and the molecular players driving Golgi fragmentation induced by amyloid-ß could be the same as those involved in the fragmentation of the Golgi ribbon during cell division (Wei and Seemann, 2017). The mechanism involves activation of cyclin-dependent kinase 5 (Cdk5) which induces the phosphorylation of the Golgi scaffold, GRASP65. The phosphorylation of GRASP65 perturbs its dimerization inducing Golgi fragmentation. Golgi fragmentation affects APP trafficking and is associated with an increase of amyloid-ß production (Joshi et al., 2014) suggesting a feedback loop with amyloid-ß and the Golgi at the center.

#### 5.2.2 Lipid and cholesterol

An imbalance in lipid and cholesterol composition has been observed in Alzheimer’s disease and could be directly linked with Golgi fragmentation [for a recent review (Pantelopulos et al., 2024)]. Cholesterol is known to modulate PI(4)P synthesis (Minogue et al., 2010) and PI4P regulates trafficking via PI(4)P effectors (von Blume and Hausser, 2019). Alterations in cholesterol metabolism has been reported to be associated with APP processing and elevated levels of amyloid-ß (Loera-Valencia et al., 2019) whereas the APP-C99 fragment (product from BACE1 cleavage of APP) can modulate cholesterol intake (Montesinos et al., 2020). An increased level of cholesterol at the plasma membrane is associated with morphological changes of the early endosomes in cultured primary neurons (Cossec et al., 2010). Interestingly, drugs to decrease cholesterol also decrease the level of secreted amyloid-β in cultured primary neurons (Simons et al., 1998) as well as reducing amyloid-β accumulation in the brains of transgenic mice (Refolo et al., 2001). However, a decrease in the plasma cholesterol levels following genetic manipulation to decrease high density lipoproteins in the plasma, has no effect on the levels of brain amyloid-β (Fagan et al., 2004). *APOE4* is known to raise levels of circulating cholesterol, and enhance amyloid pathology. *APOE4* is also associated with intracellular amyloid-β accumulation (Kuszczyk et al., 2013). Moreover, increasing the cholesterol level in neuronal culture induces the early phenotypes of Alzheimer’s disease (Marquer et al., 2014). Although the mechanism by which *ApoE4* enhances amyloid-ß production is poorly understood, cholesterol influences the properties of membranes and impacts on protein sorting events in the secretory and endocytic pathways (Bissig and Gruenberg, 2013; Lippincott-Schwartz and Phair, 2010) which could result in enhanced clustering of APP and the secretases in membranes, particularly the TGN where distinct lipid domains have been identified (Goud and Gleeson, 2010; Ramazanov et al., 2021; Tie et al., 2017), leading to increased APP processing and amyloid-ß generation.

#### 5.2.3 Cytoskeleton

Golgi fragmentation is likely to involve modification of either the microtubular and/or actin network which are wired into pathways which regulate the dynamics of the Golgi ribbon. Perturbation of the cytoskeleton (microtubules, actin, neurofilaments) can induce Golgi fragmentation and dispersal of Golgi ministacks through the cytoplasm, which will increase the distance between organelles for membrane transport and affect neuronal trafficking and signaling. Tau is associated with the Golgi membranes and microtubules in rodent neurons (Farah et al., 2006). Tau secretion after neuronal activation, is reduced following a block in Golgi dynamics using an inhibitor of Cdk5 (olomoucine) in primary cortical rat neurons whereas a slight increase of Tau secretion is observed following Golgi fragmentation induced by a knockdown of the small GTPases Rab1A (Mohamed et al., 2017). Accumulation of intracellular neurofibrillary tangles and hyperphosphorylated Tau protein can also drive perturbation of the Golgi structure and/or can also exacerbate a Golgi fragmentation already present in the cell. Golgi morphological changes have been observed in adult brain with hyperphosphorylated Tau (and in absence of amyloid-ß and no neurological disease) (Anton-Fernandez et al., 2017a). A slight increase of Golgi fragmentation is observed following the overexpression of Tau in rodent neurons and in Tau transgenic mouse, without neurofibrillary tangles formation (Liazoghli et al., 2005). More work is needed to characterize the temporal sequence of events between Tau hyperphosphorylation and Golgi fragmentation (Anton-Fernandez et al., 2017a). Also, as the Golgi is a microtubule organization center (or MTOC) (Chabin-Brion et al., 2001; Efimov et al., 2007), perturbation of the Golgi ribbon structure could, in the long term, affect the polarization of acentrosomal microtubules contributing to more permanent Golgi fragmentation and/or dispersion.

#### 5.2.4 Neuronal activity

In neurons, hyperexcitability leading to an increase of neuronal activity can induce Golgi fragmentation, which is reversible (Thayer et al., 2013). However, if the hyperexcitability is prolonged Golgi fragmentation can be irreversible and lead to impaired trafficking to synapses and defects in neuronal signaling and brain function (Thayer et al., 2013).

#### 5.2.5 Intracellular calcium and pH regulation

The accumulation of amyloid-ß can lead to an increase of the intracellular calcium which can then activate Cdk5 and the phosphorylation of GRASP65 (Joshi et al., 2014), which is known to induce Golgi fragmentation. Acute Golgi stress can induce alteration in the pH which can alter Golgi functions, reduce glycosylation and perturb Golgi structure in a more permanent way (Stanley, 2011).

Overall, we propose that the Golgi is central in an intracellular feedback loop; the initial perturbation of the somatic Golgi ribbon (arising from various sources, as described above) and the subsequent. Alterations in Golgi morphology increases amyloid-ß production, while the perturbation of membrane microdomains in turn result in loss of partitioning of APP and BACE1 in the Golgi and increased APP processing in the secretory pathway (Figures 1, 3). The morphological changes of the somatic Golgi could influence the biogenesis of the dendritic Golgi pool and, collectively, Golgi perturbations then impact on anterograde transport and delivery of membrane cargo, including signaling receptors, to the dendritic synapse and neuronal function.

## 6 Conclusion and future directions

Although a major focus for Alzheimer’s disease treatment until now has been the removal of extracellular amyloid-β aggregates and plaques, there is a plethora of data showing that intracellular amyloid-β can disrupt the function of several intracellular organelles, including organelles of the endosomal/lysosomal pathway, the anterograde transport pathway, autophagosomes and mitochondria, which result in neuronal impairment. The molecular basis for the disruption of intracellular organelles by intracellular amyloid-β and tau remains poorly understood. Only a few studies have analyzed the temporal sequence involved in the perturbation of the pathways affected by enhanced amyloid-β production and more work is required to identify the targets responsible for the compromised morphological changes of organelles and neuronal dysfunction. Cryo-electron tomography has an excellent potential to provide insights into the ultrastructural changes resulting from amyloid-β toxicity and could be used together with multi-omics to identify key targets responsible for these perturbations. A molecular understanding of the pathways involved in alterations in organelle morphology and function should provide avenues for efficient therapeutic intervention.

Here we have mainly focused on the intracellular consequences and connection between the disruption of the Golgi morphology and amyloid-β production. There are multiple pathways where amyloid-β may initiate intracellular neurotoxicity, and the key issues here are, namely, the origin of the enhanced levels of intracellular amyloid-β and the primary organelle affected. A build-up of intracellular amyloid-β could arise from either increased intracellular processing of APP, reduced rate of amyloid-β clearance or degradation and/or enhanced uptake of extracellular soluble amyloid-β. Each of these events may directly affect different organelles. For example, the familial Swedish APP mutation results in enhanced APP processing and amyloid-β production in the Golgi (Johnston et al., 1994; Wang et al., 2024b), which is likely to affect Golgi function, anterograde transport, and synaptic activity. On the other hand, Alzheimer’s disease risk factors associated with endosomal sorting result in a build-up of amyloid-β in endosomes (Bhalla et al., 2012; Tan and Gleeson, 2019). An increased cellular uptake of extracellular amyloid-β directly into endosomes within the endo-lysosomal pathways (Chia et al., 2013b) could selectively perturb lysosomal function and the autophagosome pathway (Nixon, 2007; Nixon and Rubinsztein, 2024; Peric and Annaert, 2015; Small and Petsko, 2020). Hence, the affected organelles can vary depending on the initial events responsible for the intracellular amyloid-β accumulation. It will be important to study each pathway independently and to rigorously define the sites where amyloid-β accumulates in these different scenarios and the temporal sequence of the pathways perturbed by enhanced amyloid-β production.

Emerging data indicates a heterogeneity in the pathways that seed the disease [see the review (Young-Pearse et al., 2023)] while many different genetic risk factors lead to common downstream Alzheimer’s disease pathological features. More information is required on the relationship of the individual genetic risk factors and the precise cell biological processes affected in the very early phase of the disease. In particular, the initial biochemical pathways affected during the seeding phase of the disease may differ depending on the different mutations (i.e., familial mutations of early-onset of Alzheimer’s disease, risk alleles contributing to late-onset of Alzheimer’s disease). This information is important in identifying early diagnostic markers that encompass the heterogeneity of pathways that can be affected. Progress should be now rapid in addressing this question supported by improved cell model systems.

Human iPSC-derived neurons from Alzheimer’s disease patients show phenotypic changes compared to controls. The use of human iPSC-derived neurons provides a powerful avenue to generate neurons from patients *in vitro* for use as a platform for drug discovery. Indeed, studies have begun to identify drugs from pharmaceutical compound screens which lower the levels of amyloid-β (Kondo et al., 2017). In addition, the identification of pathways responsible for the initiation of neurotoxicity has considerable potential to be exploited to identify novel druggable targets and biomarkers for treatment and detection of Alzheimer’s disease. Moreover, *in silico* drug screening against defined targets of these affected pathways can also be carried out, especially given the technology and resources of the alpha-fold protein structure databases. Such approaches provide the ability to classify Alzheimer’s disease into different categories and tailor treatment more effectively to ultimately try to curtail the early course of the disease. Also, targeting the different pathways involved in the initiation and the development of Alzheimer’s disease will be crucial to maximize the use of several drugs to inhibit amyloid-β production (Kondo et al., 2017).

A common finding in Alzheimer’s disease are morphological changes in both endosomes and the Golgi. A question arises whether these are independent or whether perturbations in one organelle impacts on the other. For example, transport between the TGN and early and late endosomes is critical for maintenance of endosomal-lysosomal homeostasis (Nakano, 2022). The pathways leading to the loss of the Golgi ribbon may result in reduced efficiency of TGN-endosome transport and thereby indirectly affect the endocytic pathway and the lysosomes. Conversely, the perturbation of the early endosomes, for example, mediated by late onset alzheimer’s disease risk alleles, may result in loss of Golgi integrity by a block in the recycling of essential regulators of Golgi trafficking back to the TGN (D’Souza et al., 2021). Also, correct localisation of glycosyltransferases throughout the Golgi stack requires machinery which is recycled from the endocytic system (D’Souza et al., 2021). Therefore, it will be also relevant to analyse how changes in the morphology of the Golgi and endocytic pathways in Alzheimer’s disease influence the glycosylation of synaptic glycoproteins receptors and the impact of such changes on the regulation of neuronal signaling. In this context, it is notable that many neuronal glycoprotein receptors have atypical N-glycans, including extension of the complex N-glycans with polysialylated oligosaccharides (Gascon et al., 2007; Mikkonen et al., 1999; Sato and Kitajima, 2021). Therefore, the challenge is to define cell networks which are altered by morphological disruption of both endosomes and Golgi.

Neuronal signaling and neuronal networking are fundamental for the functions of the brain. With the rapid development of organoid technologies, there is now the ability to expand human iPSC-derived neurons from monolayers to 3D organoids and to dissect the role of Golgi fragmentation on the organisation of neuronal networks within the complex cellular environment more relevant to the *in situ* biology. Findings from such studies will also have considerable potential to be integrated into the emerging AI field of organoid learning and memory (Smirnova et al., 2023) to bridge the gap between defects in intracellular organelles with neuronal cognition and better define the heterogeneity of the disease.

## Author’s note

Relevant literature used to write this review has been selected through Google scholar searches. When a statement was associated with a high number of articles, the authors tried to select the key publications, given the priority for studies using primary neurons and human iPSC-derived neurons and/or cited a complete and up to date review. The authors have tried to reference the main piece of data for each section and apologize if important articles are still missing.
